# Benzene-1,3-diacetic acid

**DOI:** 10.1107/S160053680802504X

**Published:** 2008-08-06

**Authors:** Mei Zhu

**Affiliations:** a Department of Biology, College of Chemistry and Biology, Beihua University, Jilin City 132013, People’s Republic of China

## Abstract

Mol­ecules of the title compound, C_10_H_10_O_4_, are connected through O—H⋯O hydrogen-bonding inter­actions into chains running along the *c* axis.

## Related literature

For related literature, see Huo *et al.* (2004 ▶); Ma *et al.* (2003 ▶).
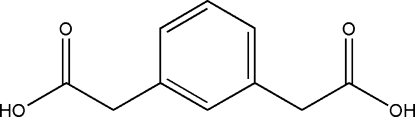


## Experimental

### 

#### Crystal data

C_10_H_10_O_4_
*M* *_r_* = 194.18
Orthorhombic, 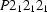

*a* = 4.9506 (9) Å
*b* = 10.0840 (17) Å
*c* = 18.576 (3) Å
*V* = 927.3 (3) Å^3^
*Z* = 4
Mo *K*α radiation
μ = 0.11 mm^−1^
*T* = 293 (2) K
0.31 × 0.21 × 0.19 mm

#### Data collection

Bruker SMART APEX CCD area-detector diffractometer
Absorption correction: multi-scan (*SADABS*; Bruker, 1998 ▶)*T*_min_ = 0.965, *T*_max_ = 0.981
5569 measured reflections
1094 independent reflections
970 reflections with *I* > 2σ(*I*)
*R*_int_ = 0.020

#### Refinement

*R*[*F* ^2^ > 2σ(*F* ^2^)] = 0.044
*wR*(*F* ^2^) = 0.121
*S* = 1.06
1094 reflections
127 parameters
H-atom parameters constrained
Δρ_max_ = 0.21 e Å^−3^
Δρ_min_ = −0.12 e Å^−3^

### 

Data collection: *SMART* (Bruker, 1998 ▶); cell refinement: *SMART*; data reduction: *SAINT* (Bruker, 1998 ▶); program(s) used to solve structure: *SHELXS97* (Sheldrick, 2008 ▶); program(s) used to refine structure: *SHELXL97* (Sheldrick, 2008 ▶); molecular graphics: *SHELXTL* (Sheldrick, 2008 ▶); software used to prepare material for publication: *SHELXTL*.

## Supplementary Material

Crystal structure: contains datablocks global, I. DOI: 10.1107/S160053680802504X/bt2762sup1.cif

Structure factors: contains datablocks I. DOI: 10.1107/S160053680802504X/bt2762Isup2.hkl

Additional supplementary materials:  crystallographic information; 3D view; checkCIF report

## Figures and Tables

**Table 1:** Hydrogen-bond geometry (Å, °)

*D*—H⋯*A*	*D*—H	H⋯*A*	*D*⋯*A*	*D*—H⋯*A*
O4—H4*A*⋯O1^i^	0.82	1.87	2.665 (3)	165
O2—H2⋯O3^ii^	0.82	1.87	2.673 (3)	165

Symmetry codes: (i) 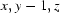; (ii) 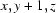.

**Fig. 1.**
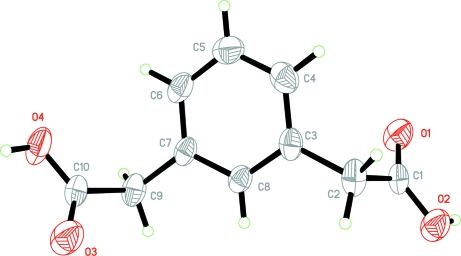
The structure of the title compound with the atomic numbering scheme. Displacement ellipsoids are drawn at the 30% probability level.

## supplementary crystallographic information

### Comment

The self-organization of small molecules with O—H···O and other weak intermolecular interactions is used to create one-, two-, and three-dimensional networks in crystalline solids (Huo *et al.*, 2004). Recently, aromatic di- or poly(carboxylic acids) have been investigated in the area of solid state and material science (Ma *et al.*, 2003). The title compound  was synthesized from benzene-1,3-diacetic acid. In the crystal, molecules of the title compound are connected  through O—H···O H-bonding interactions to chains running along the c-axis.

### Experimental

A hot aqueous solution of benzene-1,3-diacetic acid (1 mmol) was stirred until the white solids were dissolved. The clear solution was allowed to cool to room temperature and evaporated in air for 5 days. Then, colourless crystals of the title compound were obtained.

### Refinement

In the absence of anomalous scatterers Friedel pairs (714) were merged. H atoms   were generated geometrically and refined as riding atoms with O—H= 0.82Å, C_aromatic_—H= 0.93Å C_methylene_—H= 0.97Å and *U*_iso_(H)= 1.2 times *U*_eq_(C) or *U*_iso_(H)= 1.5 times *U*_eq_(O).

### Crystal data

**Table:**

C_10_H_10_O_4_	*F*_000_ = 408
*M**_r_* = 194.18	*D*_x_ = 1.391 Mg m^−^^3^
Orthorhombic, *P*2_1_2_1_2_1_	Mo *K*α radiation λ = 0.71073 Å
Hall symbol: P 2ac 2ab	Cell parameters from 1094 reflections
*a* = 4.9506 (9) Å	θ = 1.1–26.1º
*b* = 10.0840 (17) Å	µ = 0.11 mm^−^^1^
*c* = 18.576 (3) Å	*T* = 293 (2) K
*V* = 927.3 (3) Å^3^	Block, colorless
*Z* = 4	0.31 × 0.21 × 0.19 mm

### Data collection

**Table:**

Bruker SMART APEX CCD area-detector diffractometer	1094 independent reflections
Radiation source: fine-focus sealed tube	970 reflections with *I* > 2σ(*I*)
Monochromator: graphite	*R*_int_ = 0.020
*T* = 293(2) K	θ_max_ = 26.1º
φ and ω scans	θ_min_ = 2.2º
Absorption correction: multi-scan(SAINT; Bruker, 1998)	*h* = −6→5
*T*_min_ = 0.965, *T*_max_ = 0.981	*k* = −10→12
5569 measured reflections	*l* = −22→22

### Refinement

**Table:**

Refinement on *F*^2^	Secondary atom site location: difference Fourier map
Least-squares matrix: full	Hydrogen site location: inferred from neighbouring sites
*R*[*F*^2^ > 2σ(*F*^2^)] = 0.044	H-atom parameters constrained
*wR*(*F*^2^) = 0.121	*w* = 1/[σ^2^(*F*_o_^2^) + (0.0719*P*)^2^ + 0.1185*P*] where *P* = (*F*_o_^2^ + 2*F*_c_^2^)/3
*S* = 1.06	(Δ/σ)_max_ = 0.001
1094 reflections	Δρ_max_ = 0.21 e Å^−^^3^
127 parameters	Δρ_min_ = −0.12 e Å^−^^3^
Primary atom site location: structure-invariant direct methods	Extinction correction: none

### Special details

**Table:**

Geometry. All e.s.d.'s (except the e.s.d. in the dihedral angle between two l.s. planes) are estimated using the full covariance matrix. The cell e.s.d.'s are taken into account individually in the estimation of e.s.d.'s in distances, angles and torsion angles; correlations between e.s.d.'s in cell parameters are only used when they are defined by crystal symmetry. An approximate (isotropic) treatment of cell e.s.d.'s is used for estimating e.s.d.'s involving l.s. planes.
Refinement. Refinement of *F*^2^ against ALL reflections. The weighted *R*-factor *wR* and goodness of fit *S* are based on *F*^2^, conventional *R*-factors *R* are based on *F*, with *F* set to zero for negative *F*^2^. The threshold expression of *F*^2^ > σ(*F*^2^) is used only for calculating *R*-factors(gt) *etc*. and is not relevant to the choice of reflections for refinement. *R*-factors based on *F*^2^ are statistically about twice as large as those based on *F*, and *R*- factors based on ALL data will be even larger.

### Fractional atomic coordinates and isotropic or equivalent isotropic displacement parameters (Å^2^)

**Table:**

	*x*	*y*	*z*	*U*_iso_*/*U*_eq_	
O1	0.6445 (5)	1.3039 (2)	0.89747 (14)	0.0773 (7)	
O4	0.4524 (5)	0.53787 (19)	0.85467 (15)	0.0880 (8)	
H4A	0.5318	0.4677	0.8616	0.132*	
O2	1.0157 (5)	1.3432 (2)	0.83572 (13)	0.0805 (7)	
H2	0.9320	1.4113	0.8265	0.121*	
O3	0.8315 (5)	0.5825 (3)	0.79804 (15)	0.0910 (8)	
C8	0.7293 (5)	0.9447 (2)	0.85307 (14)	0.0504 (6)	
H8	0.8198	0.9542	0.8095	0.060*	
C1	0.8739 (6)	1.2698 (2)	0.87529 (15)	0.0524 (7)	
C7	0.5408 (5)	0.8441 (2)	0.86027 (14)	0.0503 (6)	
C3	0.7872 (6)	1.0315 (2)	0.90849 (15)	0.0542 (7)	
C2	0.9924 (6)	1.1399 (3)	0.89799 (18)	0.0668 (8)	
H2A	1.0900	1.1526	0.9427	0.080*	
H2B	1.1216	1.1116	0.8619	0.080*	
C10	0.5972 (6)	0.6126 (3)	0.81796 (15)	0.0557 (7)	
C9	0.4895 (7)	0.7476 (3)	0.79898 (15)	0.0626 (8)	
H9A	0.2971	0.7418	0.7897	0.075*	
H9B	0.5771	0.7794	0.7556	0.075*	
C6	0.4047 (6)	0.8332 (3)	0.92448 (16)	0.0642 (8)	
H6	0.2743	0.7676	0.9300	0.077*	
C5	0.4588 (7)	0.9185 (3)	0.98098 (17)	0.0751 (10)	
H5	0.3660	0.9098	1.0242	0.090*	
C4	0.6514 (7)	1.0169 (3)	0.97298 (15)	0.0669 (8)	
H4	0.6895	1.0735	1.0112	0.080*	

### Atomic displacement parameters (Å^2^)

**Table:**

	*U*^11^	*U*^22^	*U*^33^	*U*^12^	*U*^13^	*U*^23^
O1	0.0676 (14)	0.0528 (11)	0.1114 (17)	0.0114 (11)	0.0163 (14)	0.0152 (11)
O4	0.0761 (15)	0.0466 (10)	0.141 (2)	0.0002 (11)	0.0161 (15)	0.0274 (13)
O2	0.0756 (14)	0.0531 (11)	0.1130 (17)	0.0032 (12)	0.0200 (13)	0.0085 (12)
O3	0.0769 (16)	0.0728 (15)	0.123 (2)	0.0130 (14)	0.0221 (15)	0.0279 (14)
C8	0.0480 (13)	0.0394 (12)	0.0637 (15)	0.0030 (11)	0.0000 (12)	0.0093 (11)
C1	0.0520 (14)	0.0367 (12)	0.0684 (16)	−0.0057 (12)	−0.0130 (14)	−0.0069 (12)
C7	0.0445 (13)	0.0378 (12)	0.0686 (16)	0.0017 (10)	−0.0042 (13)	0.0142 (11)
C3	0.0507 (15)	0.0343 (11)	0.0775 (17)	0.0080 (11)	−0.0106 (13)	0.0041 (12)
C2	0.0531 (16)	0.0452 (14)	0.102 (2)	−0.0005 (13)	−0.0150 (17)	−0.0032 (15)
C10	0.0578 (16)	0.0420 (13)	0.0674 (16)	−0.0084 (13)	−0.0140 (14)	0.0039 (12)
C9	0.0645 (17)	0.0489 (15)	0.0745 (17)	−0.0043 (14)	−0.0209 (16)	0.0103 (13)
C6	0.0559 (17)	0.0465 (14)	0.090 (2)	0.0036 (14)	0.0103 (16)	0.0181 (14)
C5	0.090 (2)	0.0599 (18)	0.0756 (19)	0.0226 (19)	0.0233 (18)	0.0163 (15)
C4	0.083 (2)	0.0498 (15)	0.0682 (17)	0.0193 (17)	−0.0042 (16)	−0.0023 (14)

### Geometric parameters (Å, °)

**Table:**

O1—C1	1.256 (4)	C3—C4	1.382 (4)
O4—C10	1.244 (4)	C3—C2	1.505 (4)
O4—H4A	0.8200	C2—H2A	0.9700
O2—C1	1.258 (3)	C2—H2B	0.9700
O2—H2	0.8200	C10—C9	1.503 (4)
O3—C10	1.255 (4)	C9—H9A	0.9700
C8—C3	1.381 (3)	C9—H9B	0.9700
C8—C7	1.385 (3)	C6—C5	1.383 (4)
C8—H8	0.9300	C6—H6	0.9300
C1—C2	1.496 (4)	C5—C4	1.384 (5)
C7—C6	1.375 (4)	C5—H5	0.9300
C7—C9	1.519 (4)	C4—H4	0.9300
			
C10—O4—H4A	109.5	H2A—C2—H2B	107.6
C1—O2—H2	109.5	O4—C10—O3	123.2 (3)
C3—C8—C7	122.1 (2)	O4—C10—C9	118.2 (3)
C3—C8—H8	118.9	O3—C10—C9	118.6 (3)
C7—C8—H8	118.9	C10—C9—C7	110.2 (2)
O1—C1—O2	122.3 (3)	C10—C9—H9A	109.6
O1—C1—C2	120.2 (3)	C7—C9—H9A	109.6
O2—C1—C2	117.5 (3)	C10—C9—H9B	109.6
C6—C7—C8	118.2 (3)	C7—C9—H9B	109.6
C6—C7—C9	121.1 (3)	H9A—C9—H9B	108.1
C8—C7—C9	120.6 (2)	C7—C6—C5	120.9 (3)
C8—C3—C4	118.5 (3)	C7—C6—H6	119.5
C8—C3—C2	120.2 (3)	C5—C6—H6	119.5
C4—C3—C2	121.2 (3)	C6—C5—C4	119.9 (3)
C1—C2—C3	114.1 (2)	C6—C5—H5	120.1
C1—C2—H2A	108.7	C4—C5—H5	120.1
C3—C2—H2A	108.7	C3—C4—C5	120.3 (3)
C1—C2—H2B	108.7	C3—C4—H4	119.8
C3—C2—H2B	108.7	C5—C4—H4	119.8

### Hydrogen-bond geometry (Å, °)

**Table:**

*D*—H···*A*	*D*—H	H···*A*	*D*···*A*	*D*—H···*A*
O4—H4A···O1^i^	0.82	1.87	2.665 (3)	165
O2—H2···O3^ii^	0.82	1.87	2.673 (3)	165

Symmetry codes: (i) *x*, *y*−1, *z*; (ii) *x*, *y*+1, *z*.
